# Extramedullary plasmocytoma relapsing at differents sites: an unusual presentation

**DOI:** 10.11604/pamj.2013.14.34.1778

**Published:** 2013-01-23

**Authors:** Maryame Ahnach, Sofia Marouan, Mohamed Rachid, Abdellah Madani, Asmaa Quessar, Said Benchekroun, Meryem Quachouh

**Affiliations:** 1 Department of Hematology and pediatric oncology, 20 August Hospital, Casablanca, Morocco; 2 Department of Pathology, Ibn Rochd Hospital, Casablanca, Morocco

**Keywords:** Extramedullary plasmocytoma, primary testicular plasmocytoma, retroperitoneal plasmocytoma, recurrence

## Abstract

Extramedullary plasmacytoma (EMP) is an uncommon plasma cell neoplasm results from plasma cell proliferation and consists of monoclonal plasmacytic infiltration, without bone marrow involvement and any other systemic characteristics of multiple myeloma. EMP accounts for 3% of all plasma cell neoplasms and approximately 80% to 90% of EMP involve submucosa of the upper aerodigestive, while scrotal, dermis and retroperitoneal infiltration are very rare. There are no consensus guidelines for treatment, but EMP is highly radiosensitive, surgery may be considered for some sites, but 11 at 30% can progress in multiple myeloma. We report here an exceptional case of recurrent EMP in much localization. It's about a man 72 years old with initially testicular plasmocytoma who generalized the plasmacytic infiltration after 16 months in skin and progressively in mediastinal and retroperitoneal plasmacytoma, without any medullar and bone involvement.

## Introduction

Plasma cell (PC) neoplasms comprise a spectrum of diseases ranging from indolent conditions such as Monoclonal Gammopathy of Undetermined Significance (MGUS) to the more aggressive multiple myeloma (MM) and plasma cell leukemia. PC neoplasms are included in the World Health Organization (WHO) classification and encompass clonal PC proliferations with a wide range of clinical manifestations and behavior, which, in most cases, are associated with the production of a monoclonal immunoglobulin, or M protein, detectable in the serum and/or urine.

In the absence of disseminated bone marrow involvement, the WHO recognizes 2 types of plasmacytoma: solitary osseous plasmacytoma (SOP) and extramedullary plasmacytoma (EMP). Primary EMPs (PEMPs) are rare, constituting fewer than 5% of all PC neoplasms. Progression to disseminated PCM is infrequent, occurring in approximately 15% of cases. For unknown reasons, PEMPs have a striking propensity for involvement of the upper aerodigestive tract, while testis, dermis and retroperitoneal infiltration are very rare [1, 2]. The diagnosis of SOP and PEMP should be made only when there is a negative bone marrow result and no clinical or radiologic evidence of more widely disseminated disease or MM. There are no consensus guidelines for treatment, but EMP is exquisitely radiosensitive and external beam radiation provides excellent disease control in most cases, surgery may be considered for some sites and the 10 year overall survival rate is 70% [3].We present an unusual case of multicentric extramedullary plasmacytoma presenting on different occasions at four separate sites.

## Patient and observation

**Figure 1:**
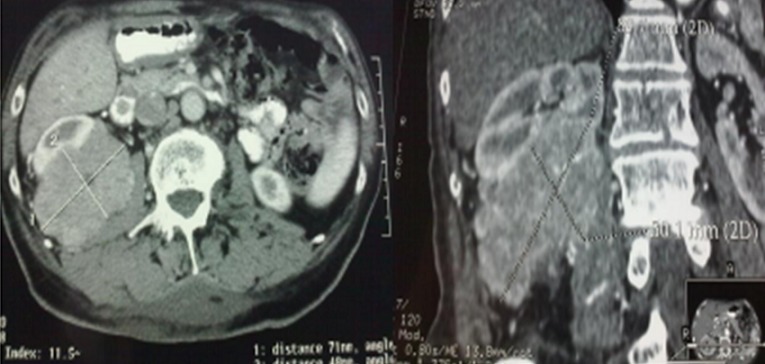
Abdominal CT scan showing the retroperitoneal localization

**Figure 2:**
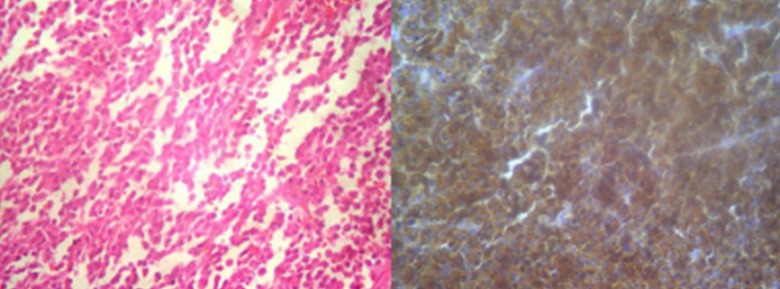
Left:H ex 40 plasmacytoid diffuse proliferation at the biopsy of retroperitoneal soft tissue Right: Immunophenotyping showing an expression of lambda chain

A 72-year old man was admitted in our department on April 2009, with a history of left testicular enlargement. There was no history of bone pain, weight loss, fatigue, fever or other systemic complaints. Physical examination revealed only a mass in the left side of the scrotum at 5cm. Full examination revealed no other abnormalities. A scrotal ultrasonography and abdominal CT scan showed a testis tumor with some small abdominal mesenteric lymph nodes. Ultrasonography of the liver, gallbladder, pancreas, spleen, kidneys, ureters, and bladder were normal. The laboratory findings were normal, including routine blood tests, urine tests, biochemical markers and LDH value. So the radical left orchidectomy was performed and the microscopic examination showed a plasmacytic infiltration with mature plasma cell and some plasmablasts; in the immunohistochemical assay, the tumor cells were diffusely and strongly positive for CD138 and lambda chains but negative for kappa chains and CD20. The pathological report concluded the diagnosis of plasmocytoma with clear surgical margins of the tumor. After this diagnosis, immunoelectrophoresis showed no evidence for hyperproteinemia or paraproteinemia. Whole body bone scan was negative and a bone marrow biopsy revealed less than 5% of polyclonal plasma cells. No Bence Jones or other M components were detected in the urine. Therefore, multiple myeloma was excluded by nuclear medicine, laboratory and histology studies. The therapeutic decision for this patient was the monitoring without any chemotherapy and radiotherapy. Sixteen months after surgery, the patient presented a subcutaneous nodule at right iliac fossa, approximately 4cm in size. The aspiration cytology of the skin lesion showed a multiple dystrophic plasma cells, and cutaneous biopsy confirmed the diagnosis of plasmocytoma. The serological examination showed an increase of the levels of free light lambda chains 66.25mg/l, and in immunoelectrophoresis we had a hypergammaglobunemia IgA at 15,3g/l. However bone marrow aspiration revealed normal patterns of cell distribution, with absence of hypercalcemia, renal insufficiency and anemia. The patient was started on chemotherapy by thalidomide 100mg/day with dexamethasone. Two months later he complained an irregular mass occupying the left side of abdomen; thoraco-abdominal CT scan showed two other localizations: mediastinal at 70x30mm and retroperitoneal 90x60mm (Figure 1). The histological examination showed similar morphology to the testicular tumor. Hence, the histological diagnosis of recurrent plasmacytoma was made (Figure 2).

Skeletal surveys did not show any pathological changes. There was no evidence of anemia, hypercalcemia or renal insufficiency. The second bone marrow biopsy was normal. Following this evolution and poor response the patient was retreated by polychemotherapy VTD (bortezomib 1,3 mg/m^2^, thalidomide100mg/j, dexamethasone 20 mg/m^2^).

On follow up, he was asymptomatic but the retroperitoneal mass still presented after 2 cycles of chemotherapy, and he developed another skin lesion at 4cm in his back. Electrophoresis of proteins found a peak gamma globulin 49.5 g/l with increasing of light chain lambda 285mg/l, and bone marrow biopsy was still normal. The patient was treated at this time by CTD (cyclophosphamide, thalidomide and dexamethasone). Evaluation after three cycles showed a good response, and the CT scan confirmed the disappearance of all masses with normalization of electrophoresis of protein. Because of the frequent recurrence and possibility of transformation into MM, the patient is still on follow up at long term.

## Discussion

Plasmocytoma is an immunoproliferative monoclonal disease of the B cell line; it's classified as non Hodgkin lymphoma [4]. EMP or extraosseous plasmacytoma are localized plasma cell neoplasm in tissues other than bone. It's a very rare subtype of plasmocytoma, and constitute approximately 3-5% of all plasmocytoma disease, the worldwide annual incidence is 3 per 100 000. The median age is 55years and 34%of all patients are male [3, 5].

According to the literature 80-90% of cases have been described in the submocosa of the upper aerodigestive tract; but it can arise any part of body in 17.8% (liver, lung, spleen, pancreas, kidney, lymph nodes, digestive tract, thyroid, heart, testis, ovary, and skin).The testis site represent 1.3% EMP, retroperitoneum 1.3% and 16.8% in epidermis [4].The first presentation in this case was a testis plasmacytoma, this site represent 0, 6% of all MM types [6]. Only 18 cases of testicular EMP were reported and many of this initially presentation progressed to MM within one year [7] or as multiple EMP in 10% of cases [8, 9].

Like our case, EMP was disseminated progressively in multiples sites: testis, skin, and retroperitoneum and mediastinal plasmacytoma; we found two other cases of recurrence EMP in the upper airway and in the breast [9, 10].

Diagnosis criteria are as follows as: Tissue biopsy showing monoclonal plasma cell histology, bone marrow plasma cell infiltration not exceeding 5%, absence of osteolytic bone lesions or other tissue involvement, absence of hypercalcemia or renal failure and low serum M protein concentration if present [11]. So the diagnosis requires multiple investigation (radiological, hematological, biochemical and histological). Radiological exams are necessary to localized the site of plasmocytoma [12] but it's the histopathologic and immunochemistry exams witch confirm diagnostic of EMP[13]. In cases of EMP, the white blood cell, renal function and calcium are in generally normal and the protein electrophoresis can show a monoclonal component in 14-25% of case [5, 14]. In our case the blood test initially was normal and serological exam revealed a gradual increase of gammaglobulin and level of light chain lambda during recurrence. The diagnosis of EMP was confirmed by many biopsy (skin and retroperitoneum biopsy), without medullary plasma cell infiltration.

There is no consensus for the treatment due to the rarity of EMP but it's depending to the degree of plasma cells extension. EMP is highly radiosensitive with 80 to 100% of patients achieving local control in low grade histology [5, 11], most of authors used radiotherapy dose 35 to 45 Gy in patient with disease > 5cm [15]. Surgery can be considered for localized type of EMP other than head neck [4, 15].

For recurrence and disseminated infiltration, adjuvant chemotherapy (regimens used for MM) may be considered after radiotherapy or surgery [3, 16]. About the patient of study, he relapse after surgery within 16 months, he was treated by poly chemotherapy VTD and CTD with good response after 5 cycles of chemotherapy. The prognosis is usually better than MM; the 10 year overall survival rate is 70% and the progression to plasma cell myeloma is infrequent about 11 to 30% within 2 years [3, 17]. Some factors affecting transformation to MM have been reported such as age (more than 63 years), and size of tumors (5 cm) [16, 18].

## Conclusion

This patient presented a very rare case of multiple EMP. According to the age, increasing of gammaglobulin and possibility of transformation to MM, this patient required a careful and long-term follow up.
